# Generation of Realistic Gene Regulatory Networks by Enriching for Feed-Forward Loops

**DOI:** 10.3389/fgene.2022.815692

**Published:** 2022-02-10

**Authors:** Erik K. Zhivkoplias, Oleg Vavulov, Thomas Hillerton, Erik L. L. Sonnhammer

**Affiliations:** ^1^ Department of Biochemistry and Biophysics, Science for Life Laboratory, Stockholm University, Solna, Sweden; ^2^ Bioinformatics Institute, St. Petersburg, Russia

**Keywords:** network biology, gene regulatory networks, gene-gene interaction, network motif structure, network generation, network simulation, benchmarking

## Abstract

The regulatory relationships between genes and proteins in a cell form a gene regulatory network (GRN) that controls the cellular response to changes in the environment. A number of inference methods to reverse engineer the original GRN from large-scale expression data have recently been developed. However, the absence of ground-truth GRNs when evaluating the performance makes realistic simulations of GRNs necessary. One aspect of this is that local network motif analysis of real GRNs indicates that the feed-forward loop (FFL) is significantly enriched. To simulate this properly, we developed a novel motif-based preferential attachment algorithm, FFLatt, which outperformed the popular GeneNetWeaver network generation tool in reproducing the FFL motif occurrence observed in literature-based biological GRNs. It also preserves important topological properties such as scale-free topology, sparsity, and average in/out-degree per node. We conclude that FFLatt is well-suited as a network generation module for a benchmarking framework with the aim to provide fair and robust performance evaluation of GRN inference methods.

## Introduction

Understanding large-scale biological relationships between genes and the proteins they encode remains a great challenge in systems biology. The wide availability of system-level expression datasets has given rise to a variety of reverse engineering methods that aim to reconstruct the hidden regulatory gene–gene and gene–protein relationships. Such relationships form a gene regulatory network (GRN) that regulates developmental processes in organisms and controls adaptation to changes in the environment (Davidson, 2010). By contrast with other networks in biological systems, GRNs are harder to validate as the interactions that occur between genes usually involve indirect interactions through biological molecules making the interaction hard to detect and quantify. The incompleteness and scarcity of ground-truth networks results in problems when evaluating the performance of methods that seek to infer GRNs from large-scale expression data (Emmert-Streib and Dehmer, 2018).

The problem of inferring a gene regulatory network from gene expression data has received significant attention. A variety of GRN inference methods are commonly used (Margolin et al., 2006; Faith et al., 2007; Friedman et al., 2010; Huynh-Thu et al., 2010; Zavlanos et al., 2011) to tackle this problem. It was also the focus of four separate Dialogue for Reverse Engineering Assessments and Methods (DREAM) challenges, with DREAM5 being the most recent one (Marbach et al., 2012). Newer, more advanced algorithms require not only expression data but also utilize additional information such as experimentally validated interactions and Gene Ontology terms (Chouvardas et al., 2016), structures of genomic datasets and network topology (Siahpirani and Roy, 2017), DNA binding domains of transcription factors, and promoter sequences of its putative targets (Kang et al., 2018), or use the iterative kernel PCR model (Iglesias-Martinez et al., 2021). Despite this, for most methods the performance on real experimental datasets remains modest (Marbach et al., 2012; Chen and March 2018; Pratapa et al., 2020).

Regardless of the method used, it is important to fairly assess its performance with respect to other methods. As some methods can only predict Boolean networks, assessment should be done in terms of binary error classification such as the number of false positives and false negatives. In addition to this, experimental information about transcriptional interactions is usually only available in the binary form. Boolean networks can only be defined by their topology, which is why it is essential to understand the structure of GRN graphs. It is also worth pointing out that most GRN inference methods can only predict a static network structure, which implies that *in-silico* generated GRNs should also possess biological stability.

While the true structure of real GRNs is usually not known, they tend to share some topological features: the scale-free property (Barabasi and Albert, 1999), where the node degrees follow a power-law degree distribution, and often have the small world property (Watts and Strogatz, 1998), and where nodes form distinct clusters in which they are connected to each other in lattice rings. These properties are different from random graphs where node degrees are normal distributed across all nodes in the system. Some attempts to simulate GRNs have been made by implementing methods that generate random (Watts and Strogatz, 1998; Mendes et al., 2003) or scale-free (Barabasi and Albert, 1999) graphs with given sets of parameters, but eventually methods based on the idea of subnetwork-selection from biological networks gained more popularity (Van den Bulcke et al., 2006). One example of this is GeneNetWeaver (GNW) (Schaffter et al., 2011), which was used to generate *in silico* networks for the DREAM challenges.

The regulatory dynamics of GRNs is shaped by network patterns that are more frequent in GRNs than in other networks (Milo et al., 2002; Shen-Orr et al., 2002) and may carry information-processing functions. These local patterns, or motifs, and do not result in emergence of specific patterns in gene expression but rather determine dynamical boundaries of the phase space of the system (Ahnert and Fink, 2016). It was suggested that some motifs could be particularly important for network dynamics and therefore become overrepresented and drive the evolution of the networks (Prill et al., 2005). Examples of how feed-forward loops are involved in such dynamics are ample, including sign-sensitive delay elements (Mangan et al., 2003), bi-phase response generators (Kaplan et al., 2008), band-pass filters (Sohka et al., 2009), and decoders of oscillatory signals (Zhang et al., 2016). Due to this, simulating a network structure that preserves the overrepresentation of motifs is of utmost importance for capturing realistic dynamics of GRNs. The idea of building gene regulatory networks by using motifs as building blocks was first introduced by Abdelzaher et al. (2015a) that hypothesized that this could be important for the evolution of GRN topology in *E. coli*.

Network inference methods aim to solve the problem of finding regulatory interactions within a set of genes. This, however, doesn’t imply that all edges in a reconstructed network represent physical binding between transcription factors and their respective targets. Gardner and Faith (2005) describe two groups of reverse-engineering algorithms. The first group seeks to identify regulators that directly control mRNA expression, and the second one is focused on identification of general regulatory interactions between different genes that may be indirect. Regardless of interaction type, simulated data should allow for exploring a wide range of network properties to evaluate inference algorithms performance. It was shown that FFLs are significantly overrepresented in experimentally validated transcriptional regulation databases (Lee et al., 2002; Milo et al., 2002). FFLs were also found to be significantly overrepresented in other databases of microRNAs and their predicted targets (Krek et al., 2005; Lewis et al., 2005) with Z-score range between 1.39 and 6.03 (Shalgi et al., 2007). Other TF-microRNA studies demonstrated that in the circuitry of gene regulation via intermediate microRNAs, in mouse and human, and the FFL motif is also enriched (Tsang et al., 2007). This suggests that FFL is an important signature of real GRNs that represent either direct or indirect interactions between genes.

In the present study the significance of 3-node motifs in four directed GRNs based on experimentally verified transcriptional interaction databases were evaluated. In agreement with previous studies (Lee et al., 2002; Milo et al., 2002; Boyer et al., 2005), it was found that the feed-forward loop (FFL) is the only motif that is overrepresented. This motivated us to develop a novel motif-based preferential attachment algorithm called FFLatt for simulating realistic structures of GRNs that are enriched with the FFL motif. The networks generated by FFLatt demonstrate structural properties that agree with biological GRNs, and have good robustness in stability analyses. Given their realistic properties, they are well suited for fair and robust evaluation of the performance of GRN inference algorithms.

## Methods

### Transcriptional Interaction Databases

Three biological databases that contain information of experimentally validated transcriptional regulation were chosen as ground-truth networks: RegulonDB (Santos-Zavaleta et al., 2019) for *E. coli* (Balaji et al., 2006), for *S. cerevisiae*, and TRRUST v2 (Han et al., 2018) for *M. musculus* and *H. sapiens* transcription factor—target regulatory relationships.

### Motif-Node Participation and Motif Enrichment

**FIGURE 1:**
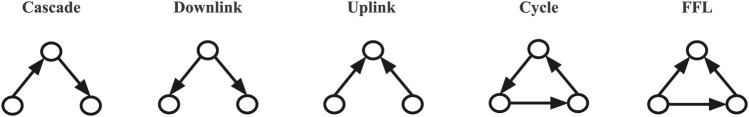
Motif collection. The five possible three-node motifs with 2 or 3 unidirectional links.

We chose to test for node-motif participation for all possible connected three-node motifs with no reciprocal links between them (Figure 1). Reciprocal links were not considered as they are very rare in the biological networks studied here. To calculate the motif-node counts, *N*_*real*_, for every node in the network we calculated the presence of a given node in all different roles of a given motif, *N(i)*. and so for a set of nodes {*1 = 1, … , M}* in the network of size *M* it could be framed as:

$$N_{real}=\overset{M}{\underset{i=1}{\sum }}N_{role1}\left(i\right)+N_{role2}\left(i\right)+N_{role3}\left(i\right)$$ (1)

**FIGURE 2:**
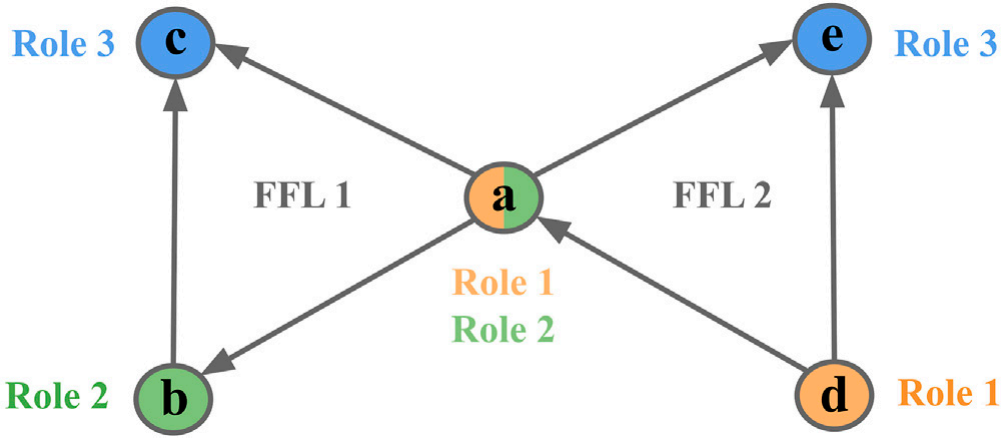
Node participation in FFL motif. An example of 3-node motif counts given on an FFL motif. Node *a* plays different roles in two FFL motifs [(**a c)** and (**d, a, and e**) respectively]. Colors represent different roles.

For example, node *a* could either participate in Role 1 (2 outgoing edges, 0 incoming), Role 2 (1 outgoing edge, 1 incoming), and Role 3 (0 outgoing edges, 2 incoming) of FFL motif 1 but at the same time participate in different role of other FFL motif 2 (Figure 2).

To test for motif enrichment, we calculated Z-score for every motif type:

$$\frac{N_{real}-\mu_{shuffled}}{\sigma_{shuffled}}$$ (2)

where *N*_*real*_ is the number of motif counts in the original network, *μ*_*shuffled*_ and *σ*_*shuffled*_ are the mean and standard deviation of motif counts in the distribution of shuffled networks. Every network was shuffled with a preserved in/out-degree for all nodes until at least 80% of edges in the original network were swapped. To calculate the mean and standard deviation of motif counts in the shuffled networks every network was shuffled 10,000 times. To ensure that the same type of nodes stay connected after shuffling, we calculated the correlations between the degree of connected nodes as weighted average nearest-neighbors degrees (Barrat et al., 2004) in the original and shuffled networks.

### Algorithm Description

**FIGURE 3:**
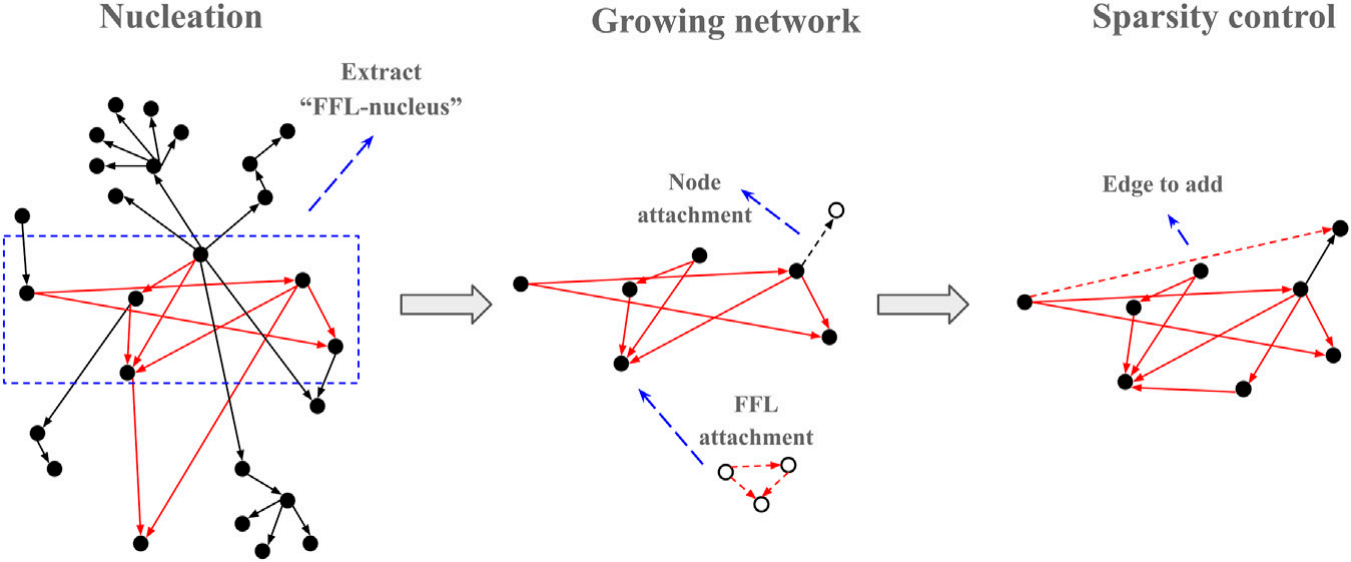
Graphic outline of the FFLatt algorithm. It starts with selecting a seed from the input network, and then iteratively grows the nucleus until the required size is reached. Finally, the sparsity of the network is adjusted according to the sparsity level.

The FFL-based generation algorithm starts with a nucleation step where an input network is used to find a subnetwork of predefined size (default 20 nodes) with all FFLs connected via shared nodes as in all analyzed networks, almost all FFL motifs share a common node with another FFL motif (Table 1). To avoid excessive parameters that could additionally control for in/out degree distribution, the *E. coli* GRN graph was used for the nucleation step. The degree distribution in the “FFL nucleus” sampled from a biological GRN was utilized by the preferential attachment rules as initial conditions to reconstruct a scale-free topology when attaching new edges and nodes to the growing network. The outline of the algorithm is presented graphically (Figure 3).

**TABLE 1:** Biological GRNs’ graph properties.

Organism	# Of nodes	% Of nodes that participate in FFL motifs	% Of FFL motifs sharing nodes with other FFLs	Sparsity	In-degree	Out-degree
*E. coli*	1,917	37.4	99.1	2.328	1.106	1.222
*S. cerevisiae*	4,441	27.0	100	2.899	1.421	1.477
*M. musculus*	2,862	31.5	99.7	2.643	1.274	1.369
*H. sapiens*	2,456	34.7	99.9	2.944	1.364	1.580

**FIGURE 4:**
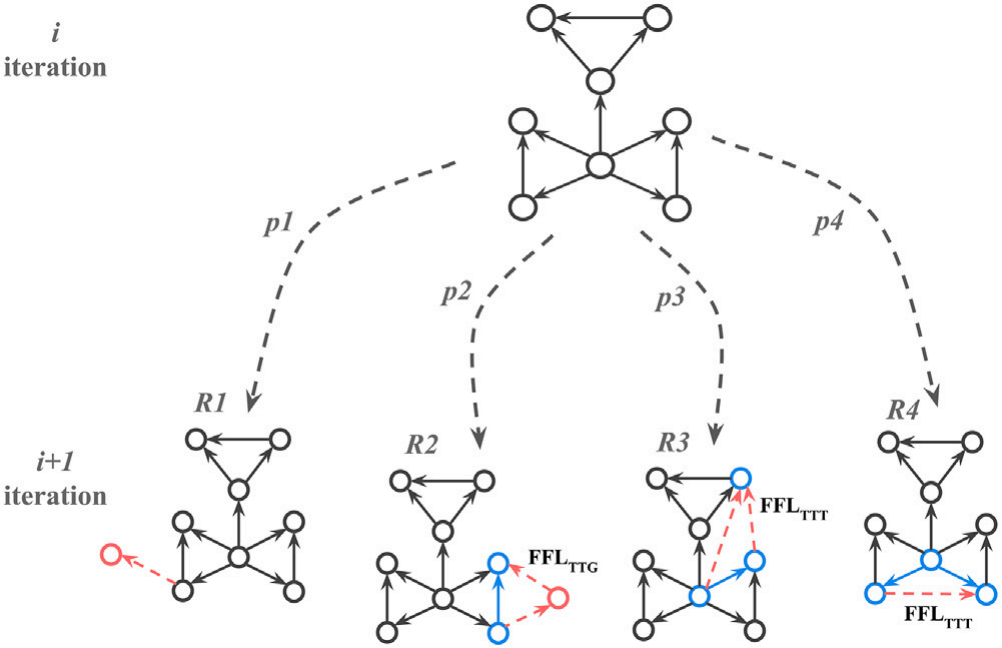
Attachment rules that create FFL motif enriched network; *p1*, *p2*, *p3*, and *p4* correspond to probabilities for choosing rule *R* at the next iteration while growing network. FFL_TTG_ and FFL_TTT_ correspond to different FFL motif types, where G or T (Gene or Transcription factor) indicate whether a participating node has only incoming edges (G), or at least one outgoing edge (T). The red dotted arrows here show new edges added to the network and the solid blue arrows show edges participating in the new FFL motif with the new edges.

Once the substrate is selected the algorithm adds nodes and edges iteratively such that at every iteration, a candidate node is selected with a random uniform probability. Once selected, one of the four attachment rules (R1, R2, R3, and R4) is applied (Figure 4) based on four predetermined probabilities (*p1, p2, p3*, and *p4*) that add up to 1. The iterations are repeated until the required number of nodes in the network is reached.

If the random float number *r1* is less or equal to *p1* then R1 is picked. For the R1 rule we applied the modified preferential attachment algorithm from Abdelzaher et al. (2015a) with a power-law kernel:

$$P\left(g\right)=\frac{K_{g}^{\gamma }}{\overset{n}{\underset{i=1}{\sum }}K_{i}^{\gamma }}$$ (3)

where *K*_*i*_ denotes node-degree connectivity, *P(g)* is the probability that a new node will be connected to existing node *g*, and *ɣ* is a parameter that controls the shape of the out-degree distribution.

If *r1* is greater than *p*_*1*_ then one of the motif-based preferential attachment rules (R2, R3 or R4) is applied, and so *1-p*_*1*_ corresponds to the desired percentage of nodes that participate in FFL motifs. For R2-R4 rules, one of the already existing FFL motifs is picked based on it’s connectivity with the others.

Once the candidate motif and rule are chosen, a new random float number, *r2*, is generated. If 0 < *r2* ≼ *p*_*2*_ *,* the R2 rule is applied. In that case, two new edges and one new node will be added to the existing node so the new FFL motif is formed. If *r2 > p*_*2*_, one of the R3 or R4 rules is selected with equal probability. For the R3 rule, two edges are added to nodes in existing FFL motifs to create a new FFL motif. For the R4 rule, and one edge is added between nodes in two existing FFL motifs to create a new FFL motif. If R2 is applied, it creates an FFL motif where one node has only incoming edges. If R2 or R3 is applied, it creates an FFL motif where all participating nodes have at least one incoming and one outgoing edge. See Figure 4 for details.

All nodes have to have an out-degree smaller or equal to a threshold *K*_*max*_ after which no new outgoing edges are added. If the candidate motif doesn’t satisfy the conditions for a chosen FFL attachment rule, another candidate motif picked and this is repeated until a motif is found that meets the rule conditions. If a new motif is created, the library with FFL motifs is updated.

When the desired network size is reached, the algorithm adjusts the sparsity (average number of connections per gene) until it reaches the set sparsity level in terms of average links per node. If the network is too dense, edges are selected for removal based on out-degree node connectivity so that an edge is proportionally more likely to be removed if it is attached to a node with a high out-degree. If the network is too sparse, edges are added to nodes selected proportionally to their out-degree connectivity, connecting them to randomly selected nodes. When network generation is completed, the network is saved as an unweighted directed graph.

### Network Generation

For network simulation comparison five algorithms were chosen: FFLatt (developed in present study), GeneNetWeaver (GNW; Schaffter et al., 2011), NetworkX directed scale-free graph algorithm (NetworkX; Hagberg et al., 2008), and sparse uniformly distributed random matrix with and without allowing for feedback loops in the network (DAG and RandG; Guo and Amir, 2021). DAG and RandG matrices were binarized by setting all non-zero elements equal to 1. The NetworkX graph algorithm was modified to control for sparsity as the FFLatt algorithm does, i.e., edges are added to or removed from nodes proportionally to their out-degree node connectivity. For network generation of different sizes with FFLatt, the set of transcriptional interaction graph properties estimated from the *E. coli* transcriptional interaction network (Table 1) was used. For each organism, the number of nodes that participate in FFL motif was used to set *p1*, with *p2* equal to (1-*p1*)*0.9, and *p3*=*p4*=(1-*p1*)*0.05 respectively. For network generation of different sizes with other algorithms (except GNW), only network size and sparsity parameters were taken into account as only controllable parameters available. For network generation/subselection with GNW the following (default) parameters were used: *-random-seed*, *--greedy-selection, --keep-self-interactions* as well as the size of the subtracted network.

When mimicking the *E.coli* transcription network model, all three-node cycles were disrupted, by removal of one edge, as they are absent in the target network. The removal was done by deleting the outgoing edge of the node with the highest out-degree and an edge was instead attached to a random node with a probability based on the connectivity of each node.

To mimic the complete three-node motif profile in biological GNRs in which non-FFL motifs are depleted, an optional motif depletion step can be executed. Here all three-node cycles are converted to FFL motifs by swapping the direction of one of the edges. In addition, up to one tenth of the cascades that do not share edges with FFL motifs were used to create new FFLs by adding an edge. The total number of edges that was used for motif conversion was taken into account when adjusting the network sparsity.

For stability analysis, self-loops (if any) were removed from network graphs generated with above mentioned algorithms before applying the stability analysis model.

### Stability Analysis Model

To measure the stability of a network, i.e., how a network graph structure affects the dynamical stability of a gene regulatory interaction model, we utilized the model developed by (Guo and Amir, 2021) that explores how the dynamics of protein and mRNA concentrations control the transcriptional regulation. The model allows for multiple proteins acting on the same gene, and is defined by the authors as:

$$g_{i}\left(\vec{c}\right)=g_{i0}+\underset{j}{\prod }\left(1+\gamma_{ij}f_{ij}\left(c_{j}\right)\right)$$ (4)

where *g*_*i*_ and *g*_*i0*_ is the effective gene copy number of gene *i* with and without input of other genes respectively, *c*_*j*_ is the concentration of transcription factor *j*, and *γ*_*ij*_ relates to the strength of the regulation of gene *i* by *c*_*j*_. The functional relationship between the transcription factor and target gene, *f*_*ij*_, is modelled as a sigmoid Hill function:

$$f_{ij}\left(c_{j}\right)=\frac{c_{j}^{h}}{K_{ij}^{h}+c_{j}^{h}}$$ (5)

where *h* is the saturation binding coefficient, i.e. the number of proteins required for saturation of binding to DNA, and K is the protein concentration threshold needed to produce a significant increase in mRNA.

The process of gene expression could be described as coupled dynamics of protein and mRNA concentrations. It was shown that in yeast (Zhurinsky et al., 2010) and mammalian cells (Schmidt and Schibler, 1995), the RNA polymerase concentration limits the transcription of mRNA, and the number of ribosomes limits the process of translation. The general transcription model (4) that connects transcription rate of gene *i* and the number of RNA polymerases can then be described as:

$$\frac{dCm_{i}}{dt}=k_{m}\phi_{i}\left(\vec{c}\right)n-C_{mi}k_{p}c_{r}-\frac{C_{mi}}{\tau }$$ (6)

$$\frac{dc_{i}}{dt}=k_{p}c_{r}\left(\frac{C_{mi}}{C_{mT}}-c_{i}\right)$$ (7)

where *n* is the total number of RNA polymerases, $C_{mi}$ is the mRNA concentration of gene *i*, $C_{mT}$ is the concentration of all mRNAs, *ϕ* is the gene allocation fraction of $g_{i}\left(\vec{c}\right)$ controlled by RNA polymerases active on gene *i*, *k*_*m*_ is the transcription rate of RNA polymerase, *k*_*p*_ is the translation rate of the ribosome, *c*_*r*_ is the ribosomal concentration, and τ is the degradation rate difference between proteins and mRNA.

We assume that mRNAs degrade much faster than proteins, and as suggested by (Guo and Amir, 2021) we can set $\frac{dCm_{i}}{dt}$ ≈ 0 to neglect fast dynamics aiming to simplify the model. By substituting $C_{mi}$ from 6 into 7, the dynamics of transcription factors concentrations can be simplified as:

$$\frac{dc_{i}}{dt}\approx k_{p}c_{r}\left(\phi_{i}\left(\vec{c}\right)-c_{i}\right)$$ (8)

In such case, the stability of a steady-state in the dynamical model is dependent on the Jacobian matrix *A* of size *N*x*N*:

$$A=k_{p}c_{r}^{ss}\left(M-I\right)$$ (9)

where $c_{r}^{ss}$ is the steady-state ribosomal concentration, *M* is the gene-gene interaction matrix that consists of *γ*_*ij*_ weights of the regulation, *I* is the identity matrix, and *N* is the number of genes in the system. The system is stable if the maximal real part of all eigenvalues of *M*, *λ*_*M*_, is smaller than 1, i.e., the real part of all eigenvalues of *A* are negative. As the imaginary part of the eigenvalues is ignored, both oscillatory systems and systems without oscillations around the steady state are considered to be stable.

In contrast to random matrix theory (May 1972) or the generalized models (Gross and Feudel, 2006; Gross et al., 2010), the Jacobian matrix here is not a random matrix nor approximated through studying system bifurcations. In the Guo and Amir model it is derived by applying a knowledge-driven modelling approach which we find convenient for such a well-studied biological process like transcription. We applied this model to all network graphs simulated with different algorithms. Each graph, in a form of adjacency matrix, was supplied as a binary interaction matrix. For each replicate of a different size generated with a given algorithm, we repeated assigning the network graph with link strengths 10 times. To focus on the effect of the GRN structure and FFL content on stability, we forced the distribution of link strengths of all GRNs to be similar. This was done by randomly setting half of the links in the binary interaction matrix to be upregulated and the other half downregulated (setting max (*γ*_*ij*_) and min (*γ*_*ij*_) to 1.5 and −1.5 respectively as boundaries of a normal distribution). In every trial, we first numerically solved for the ribosomal concentration $c_{r}^{ss}$ with which the system reaches its non-zero steady state with Eq. 8. Given $c_{r}^{ss}$ , *A* was found such that it only has negative real part eigenvalues using Eq. 9 by optimizing *M*, and the highest eigenvalue in *λ*_*M*_ from this solution was compared across networks of different sizes.

## Results

### Feed-Forward Loop is the Only Enriched Three-Node Motif in Biological Gene Regulatory Networks

Of all possible 3-gene network motifs with 2 or 3 unidirectional links, we found a strong enrichment relative to shuffled networks of the FFL motif in the networks studied here, which are networks that mainly capture transcription factor to target interactions (Supplementary Table S1). This was previously shown for *E. coli* (Milo et al., 2002) and *S. cerevisiae* (Lee et al., 2002). We also found that the cascade, uplink, and downlink motifs were consistently and significantly (*p*-value < 0.05) depleted in all four target networks. To ensure that the shuffling procedure produced topologically similar networks, we verified that the distribution of correlations between the degree of connected nodes was similar for the original and shuffled networks (Supplementary Figure S1).

All depleted motifs are 3-node motifs with two edges (Figure 1), and these have previously been shown to be significantly depleted in other biological networks, for instance in a protein structure network and a human brain functional network (Mirzasoleiman and Jalili, 2011). However, how the depletion of these motifs contributes to the function of the gene circuitry, and how it relates to the evolution of gene regulatory networks, remains to be answered.

We found that FFL is the only enriched motif, and this was observed in all analyzed networks (Supplementary Table S1). Almost all FFL motifs share a common node with another FFL motif, as this fraction ranges from 99.1% in the *E. coli* GRN to 100% in *S. cerevisiae* (Table 1). The fraction of nodes that participate in FFL motifs ranges from 27 to 37.4%. This inspired us to develop a GRN generation algorithm that attaches nodes to form connected FFL motifs at a high rate. For each GRN we also calculated the average number of edges per node, here referred to as sparsity, and average in- and out-degrees, and these properties were also used as targets for the algorithm.

Each regulatory interaction in the FFL motif can be either positive or negative, i.e., activating or inhibiting, resulting in eight different types that can act as e.g. accelerators, delay-generators or pulsers (Mangan and Alon, 2003), resulting in different dynamics of gene circuits. Given the wide variety of FFL types and their importance to GRN dynamics, an unsigned *in silico* GRN graph needs a large number of FFLs to accommodate these. A combination of the eight signed types of FFL motifs will in turn reflect a realistic flow of GRN circuits.

**FIGURE 5:**
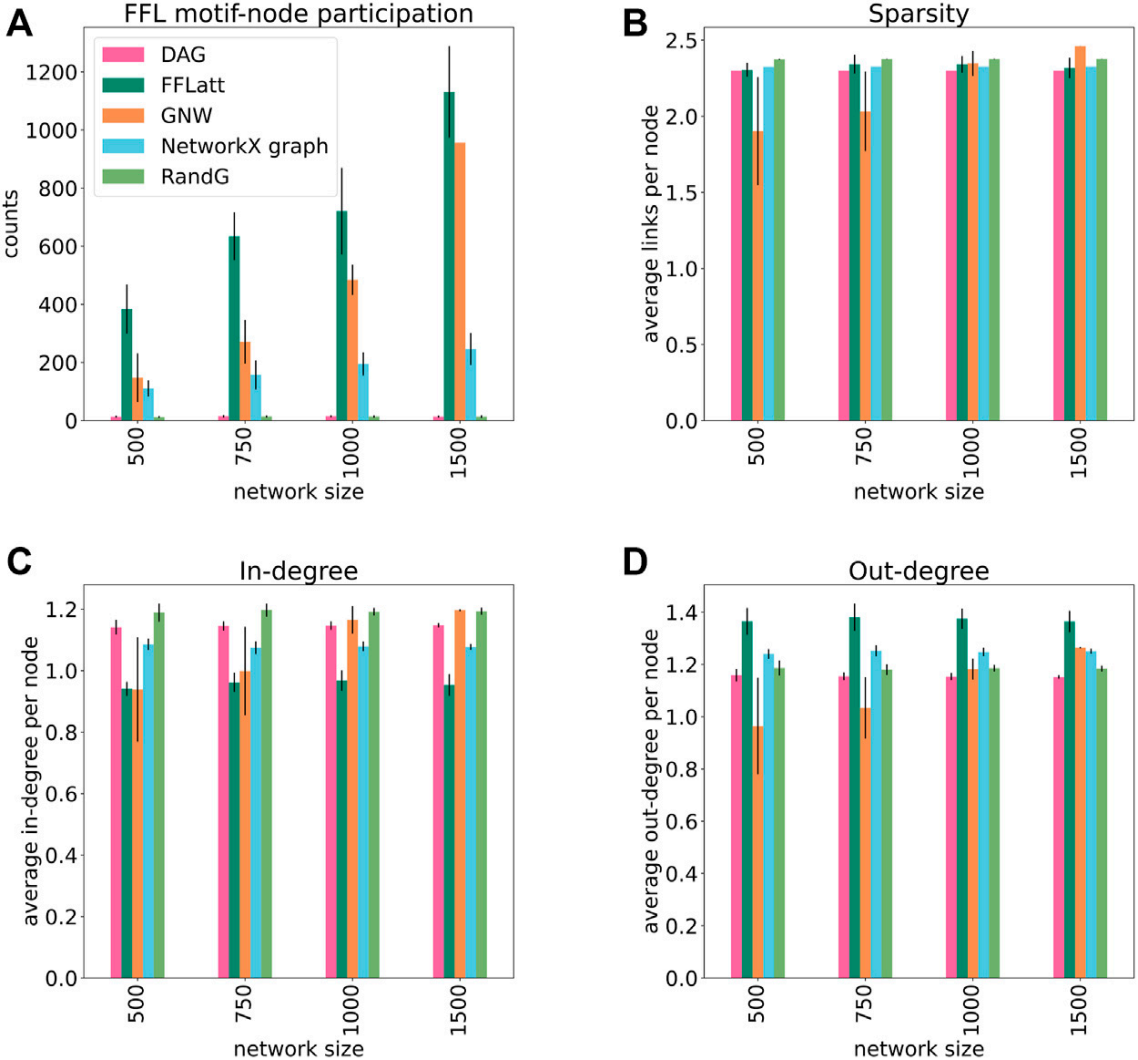
Topological properties of simulated networks (*E. coli*). FFL motif node participation, average sparsity, in- and out-degree distribution in simulated networks. For FFL-motif node participation counts, up to three participations for each node were allowed (in different roles). Each data point was calculated as the average of ten different replicates of each network size. Error bars represent standard deviation.

We generated a set of GRNs of different sizes from 500 to 1,500 nodes, 10 replicates for each size, using five different algorithms: FFLatt, GNW, NetworkX graph, RandG, and DAG. For each algorithm we analyzed four properties of their GRNs: the number of nodes that participate in FFL motifs, network sparsity, average in- and out-degree within the network. We repeated these simulations for all four organisms, as they have different graph properties. The results for *E. coli* are shown in Figure 5, and for the other organisms in Supplementary Figures S2, S3, and S4. Each organism-related GRN was used to set the topological parameters in the GRN simulated by FFLatt as described in Methods.

To assess the accuracy of GRN inference algorithms, the topological parameters such as in- and out-degree distribution and sparsity should be controlled when simulating data for benchmark analysis. We found that sparsity as well as out-degree of artificial networks generated with the subnetwork selection based GNW algorithm deviates considerably from the target networks for *E. coli* in sizes 500 and 750 (Figures 5B,D), for *S. cerevisiae* in size 500 (Supplementary Figures S2B, S2D), and in all sizes for *M. musculus* and *H. sapiens* (Supplementary Figures S3B, S3D, S4B, and S4D). While this alone does not indicate a poor performance of the GNW algorithm, it does advocate for the necessity of network generation algorithms to control topological parameters.

**FIGURE 6:**
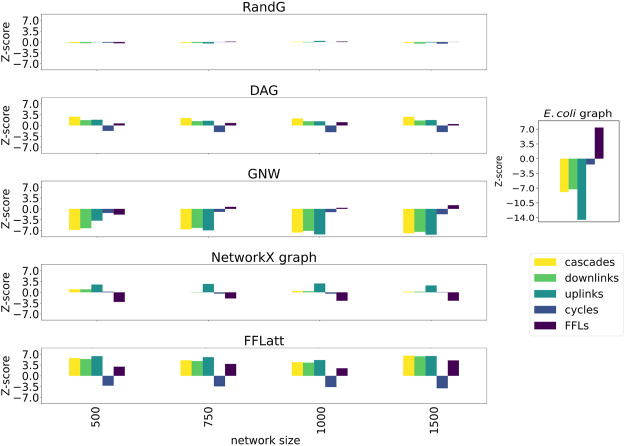
Motif enrichment analysis of 3-node network motifs in simulated networks (*E. coli*). For networks generated with GNW, the *E. coli* RegulonDB (Santos-Zavaleta et al., 2019) database was used. For networks generated with FFLatt, we used the graph properties for *E. coli* specified in Table 1. RandG is a random assignment of links and DAG is the same with cycles removed. NetworkX graph GRNs are scale-free. For RandG, DAG, and NetworkX graph GRNs we used the *E. coli* network sparsity.

More importantly, when subsetting networks from biological GRNs with the GNW algorithm, we obtained a significant underrepresentation of FFL motifs in sizes 500, 750, and 1,000 for *E. coli* (Figure 5A) in comparison with FFLatt networks. Similar results were obtained for GRNs of other organisms (Supplementary Figures S2A, S3A, and S4A). To confirm and extend these findings, we performed motif enrichment analysis on the simulated networks as well as on biological GRNs (Figure 6; Supplementary Table S1). This showed that FFL motifs are not significantly overrepresented in GNW networks, but they are highly significantly enriched in the *E. coli* GRN (Z-score 7.4). In networks generated with other algorithms, the FFL motif was also not significantly overrepresented, with the exception of FFLatt whose networks were significantly enriched with Z-scores between 2.95 and 4.98. By default, FFLatt does not deplete other 3-node motifs, and but this is possible with an optional motif depletion step. We explored how this step in combination with various parameter values can mimic the complete 3-node motif distribution profile with the FFL motif enriched, and all other motifs depleted (Supplementary Table S2).

### Topology, Motif Composition, and Network Stability

In biology, random matrix theory, that seeks to understand the properties of matrices with randomly drawn elements, is known from R. May’s research on the stability of large biological systems (May 1972). He demonstrated that the stability of a large ecological system depends on satisfying the following inequality:

$$1>\alpha \sqrt{nC}$$ (10)

where *α* is the average interaction strength, *n* is the number of species, and *C* is the density of interactions between them. Therefore, the larger a system gets the more unstable it becomes unless the sparsity and/or interaction strengths are scaled down accordingly. May’s approach has been proven to be highly valuable to other biological networks (Aljadeff et al., 2015), including those that aim to describe gene regulations (Prill et al., 2005; Stone, 2018).

**FIGURE 7:**
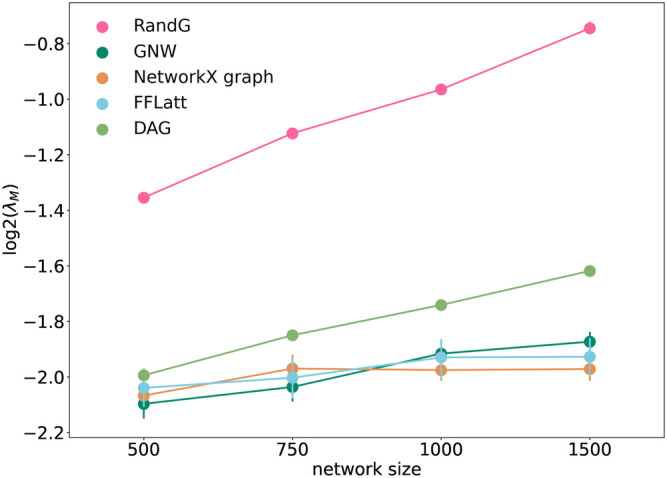
Stability of randomly wired simulated network graphs. *λ* is the lowest eigenvalue of the interaction matrix *M*. Each data point was calculated as the average of ten different repeats of overlaying links chosen randomly with strengths from a standard distribution, with corresponding semi-transparent areas indicating the 95% confidence interval.

It was earlier suggested that motif composition contributes to fault-tolerance in transcriptional networks (Roy et al., 2020). To test if the structural composition is important for stability in artificially generated networks, we analysed the stability of the five network models using the method by Guo and Amir (2021). As expected, all GRNs with fixed sparsity and interaction strengths became more fragile when increasing in size. We found that GRNs with different motif profiles demonstrated different levels of network stability (Figure 7). The RandG GRNs that were neither enriched nor depleted with any 3-node motifs (Figure 6) were far less stable than the other ones. The DAG GRNs which are generated like RandG GRNs but without cyclic motifs were more stable but still considerably less stable than NetworkX, GNW, and FFLatt GRNs. We note that NetworkX, GNW, and FFLatt GRNs have different network motif abundances, such as either depleted or enriched FFL motifs, and yet they show similar stability. The abundance of the FFL motif alone therefore does not seem to be a major factor for network stability, which is congruent with previous findings about non-importance of the FFL motif to system robustness under random node failure test (Abdelzaher et al., 2015b).

**FIGURE 8:**
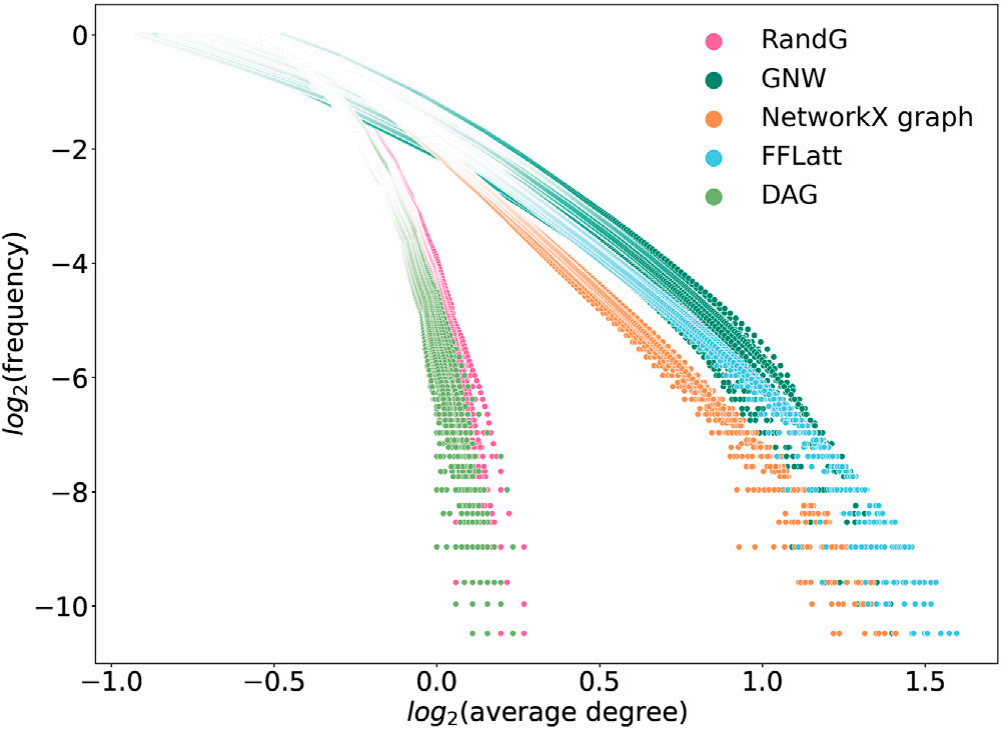
Degree distributions in simulated networks generated by different algorithms. GRNs of sizes 500, 750, 1,000, and 1,500 were used, ten of each size. A power-law distribution should generate a straight line.

We note that the two lines that represent size-dependent stability of DAG and RandG GRNs have a steeper slope than the other three. This means that as the GRN increases in size, DAG and RandG GRNs become less stable faster than the other three. To find a reason for this, we analyzed the degree distribution of the GRNs. Since RandG and DAG networks are sparse uniformly distributed random binary matrices, their degree distributions do not follow the power-law and therefore they are not scale-free (Figure 8). This suggests that a scale-free topology which has been previously found to be central for creating a robust system, protecting the GRN from random mutations (Greenbury et al., 2010), can in fact help gene regulatory systems to reach a stable state after perturbation.

## Discussion

Here we present a new algorithm, FFLatt, for generating realistic directed GRN graphs to enable more accurate and authentic performance evaluation of GRN inference methods. The novelty of the presented algorithm is that it generates networks with boosted FFL motifs, which are known to be important for network dynamics. Besides being enriched with the FFL motif, the resulting GRN graphs generated with FFLatt exhibit topological properties similar to experimentally validated biological GRNs.

We show that the motif profile and topological properties of FFLatt network graphs demonstrate a biological stability comparable with other models, such as the NetworkX and GNW algorithms. It is particularly important for network inference methods working with steady-state gene expression data as many of them, for instance Least-Squares with Cut-Off (LSCO; (Tjärnberg et al., 2013), LASSO (Tibshirani, 1996; Friedman et al., 2010), LASSO-VAR (Larvie et al., 2016), and GENIE3 (Huynh-Thu et al., 2010) aim to infer a stable static network from steady-state data. To summarize, the FFLatt graph generation algorithm provides an opportunity to simulate biologically meaningful network graphs that can be wired with realistic biological dynamics.

We also noted that the FFLatt networks were enriched with three other motifs: uplinks, downlinks and cascades whereas in GNW networks and biological GRNs these motifs are usually depleted. Sorrells and Jonhson (2015) suggested that in biological GRNs, FFL formation proceeds through a non-adaptive rewiring of gene regulatory regulation which could explain how the abundance of FFLs and the depletion of uplinks, downlinks, and cascades is coupled. The algorithm can be run to allow for depletion of other 3-node motifs while growing the network. However a reason that such depletions are important for network dynamics is yet to be found. A thorough search of the relevant literature did not yield in related articles. We also could not find evidence that different three-node motif profiles affect network stability. NetworkX, GNW, and FFLatt motif profiles are fairly different yet they demonstrated comparable stability across different sizes. While being out of scope for this study, it remains an interesting question how the composition of more complex and higher-order structures known to be present in GRNs (Benson et al., 2016; Gorochowski et al., 2018) could contribute to stability of the system.

In this article we focus on the proof of concept of the FFL attachment algorithm to demonstrate its necessity and feasibility. However, to increase model performance, it could be extended with other parameters. For example, to better capture “small world” (Watts and Strogatz, 1998) structural properties that are known to be present in biological networks, one parameter could be a desired number of biological modules so that within each module the connectivity is higher than in between them. The clustering algorithm should however be biologically motivated so that the connection between modular graph structure and expression dynamics is clear.

Despite a continued uncertainty of how structural properties and functional modularity of GRNs relate to each other, some patterns such as FFLs are known to be key signatures of transcriptional regulation networks. Here we developed a novel algorithm that generates biologically realistic structures of large artificial gene regulatory networks with controlled size, sparsity, topology, and number of FFLs. The implementation executes with reasonable runtimes (Supplementary Figure S5). FFLatt graphs are binary and can thus assume a wide range of dynamical structures with signed strengths. They could be used as input to already established tools based on Hill function kinetics such as GNW, which allows for knock-out and knock-down perturbation designs when generating expression data, and some control of the number of nodes, including the number of transcription factors, based on a user-defined input network. To generate expression data it utilizes a non-linear ordinary differential equations (ODE) model for gene expression, and stochastic differential equations (SDEs) for molecular noise generation. Potentially, they could also become a part of future deep learning frameworks that aim to model gene expression from DNA sequence (Zrimec et al., 2020; Avsec et al., 2021). In such frameworks, FFLatt networks could be used as a deep learning model constraint to incorporate prior knowledge of each node participation in FFL motifs. As a result, we believe that it will contribute to future development of benchmarking tools that could fairly and accurately evaluate the performance of GRN inference methods.

## Data Availability

The original contributions presented in the study are included in the article/Supplementary Material. The source code of the algorithm is available at https://bitbucket.org/sonnhammergrni/fflatt.
