# Supplementary figures and images for: Generation of Realistic Gene Regulatory Networks by Enriching for Feed-Forward Loops
Source: Front Genet. 2022 Feb 10;13:815692. doi: 10.3389/fgene.2022.815692 (PMC8872634; doi:10.3389/fgene.2022.815692)

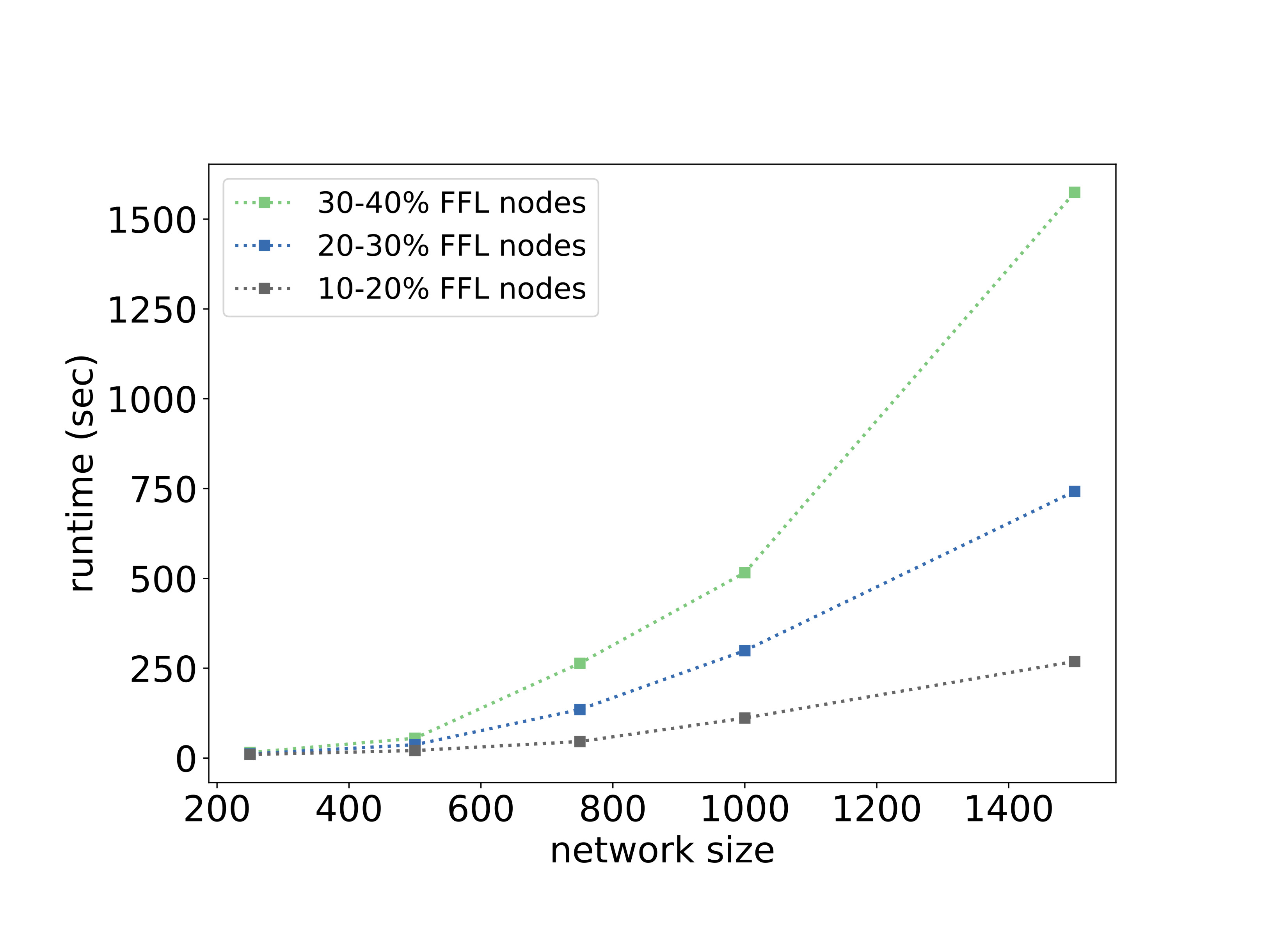

Supplement: Supplementary file 1 [file Image5.jpg]

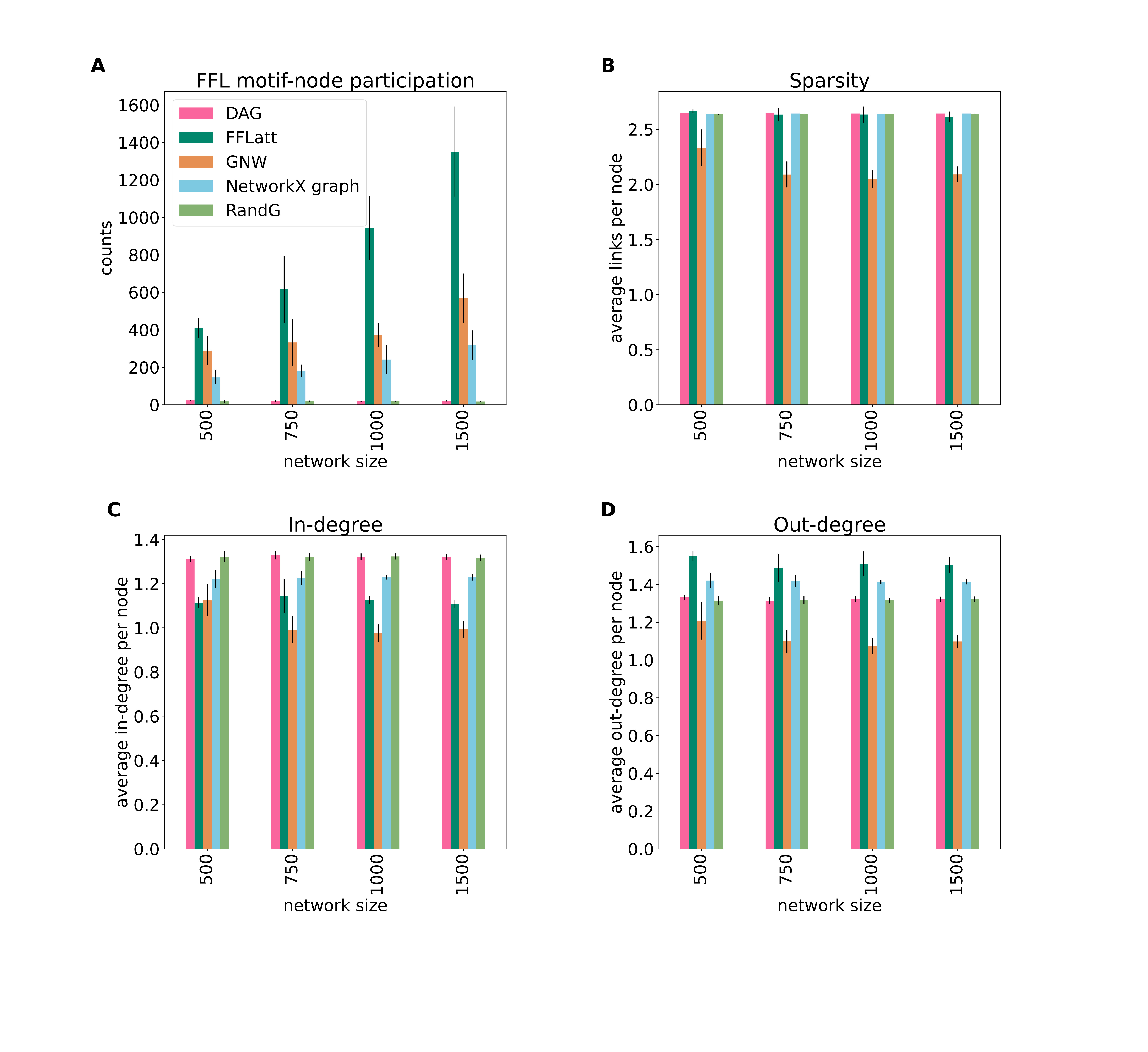

Supplement: Supplementary file 2 [file Image3.JPEG]

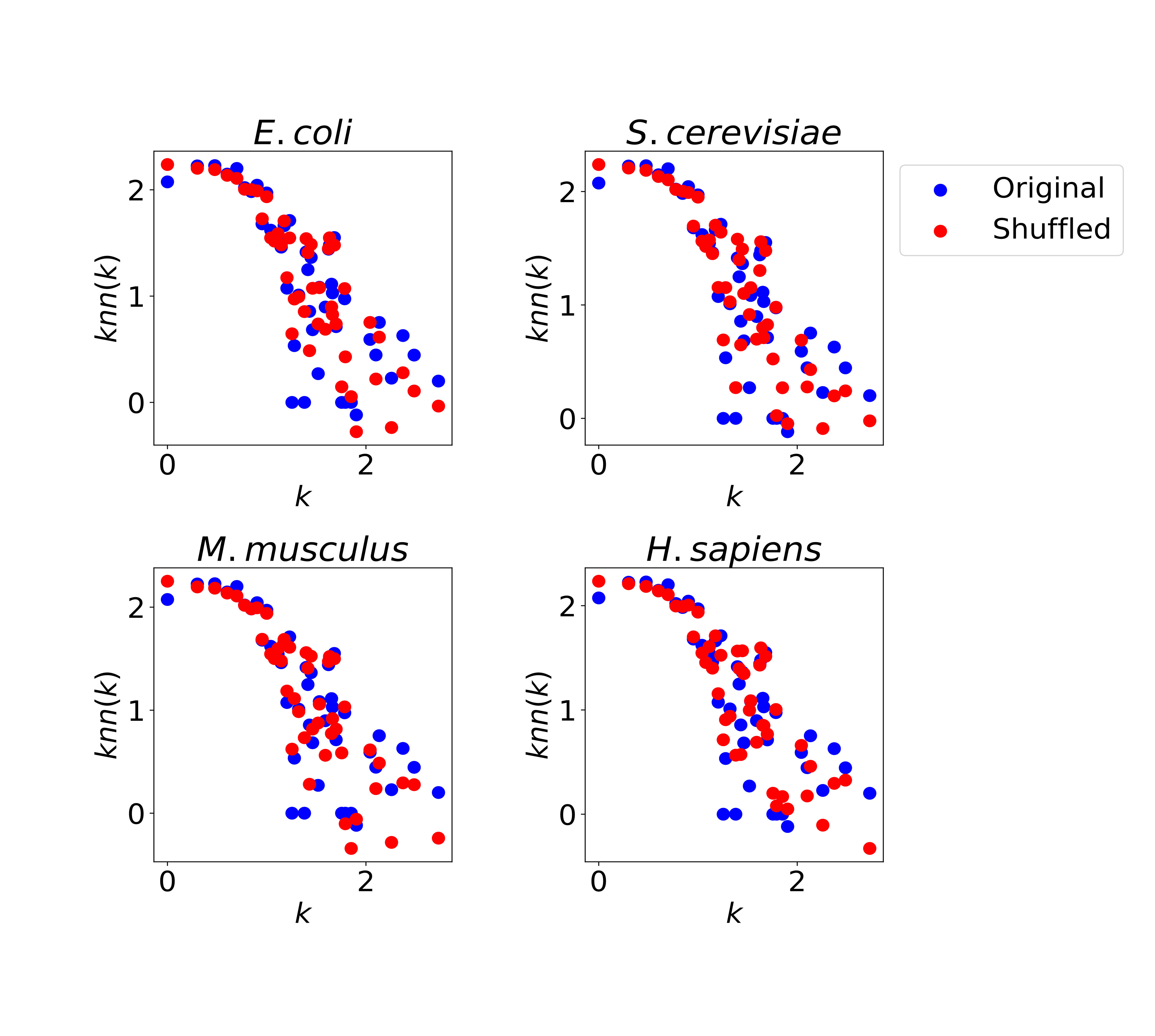

Supplement: Supplementary file 3 [file Image1.JPEG]

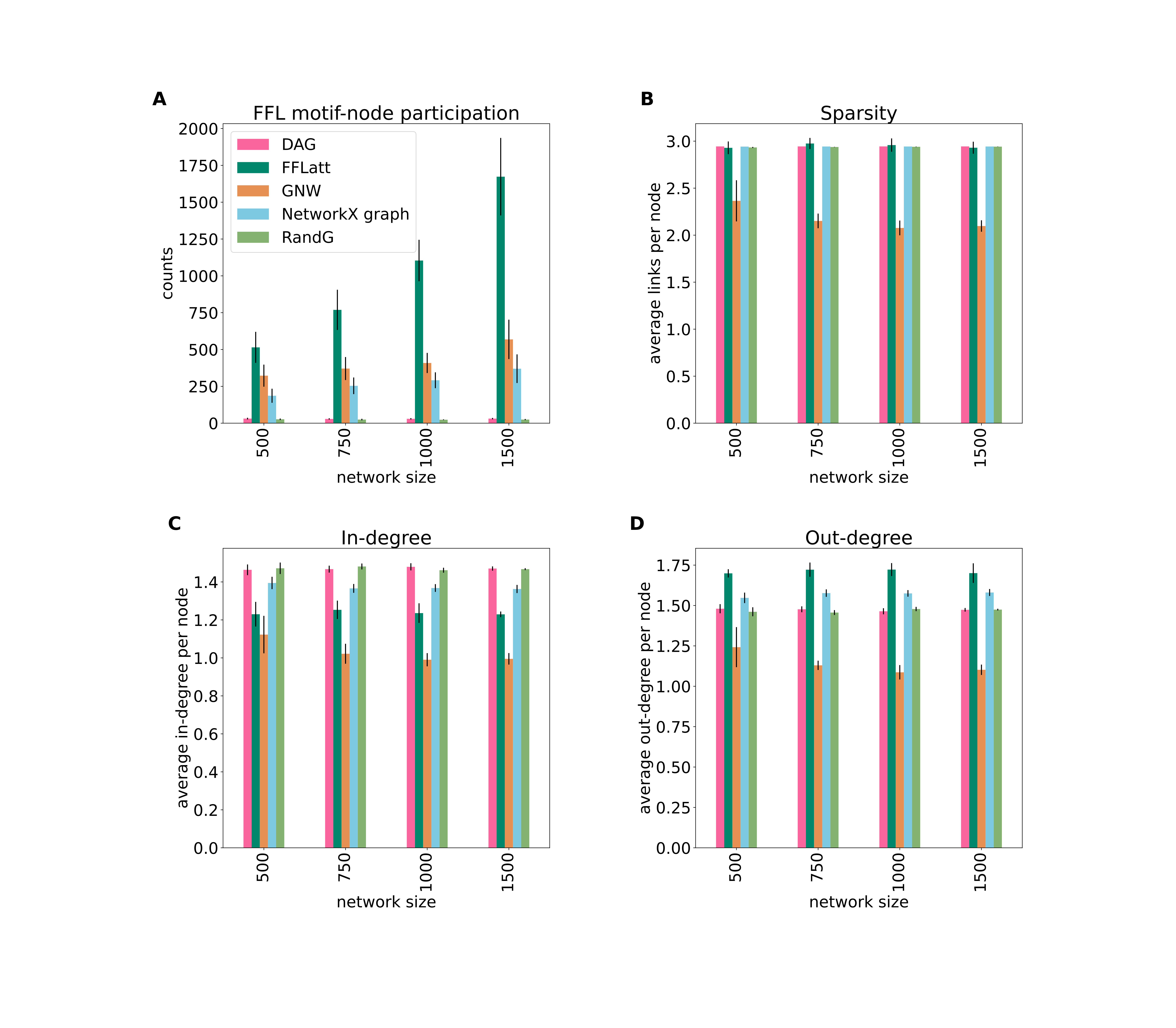

Supplement: Supplementary file 4 [file Image4.JPEG]

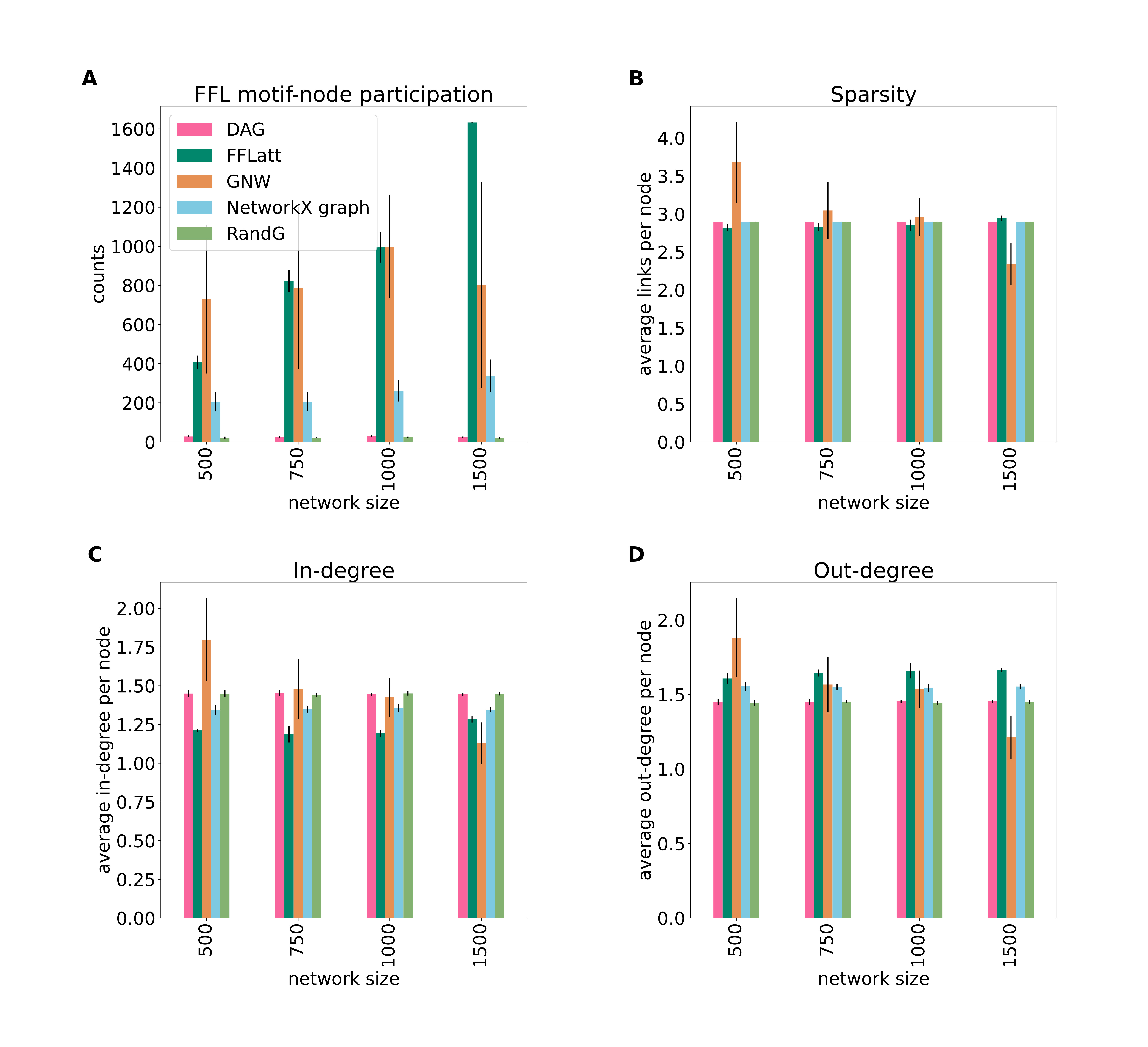

Supplement: Supplementary file 5 [file Image2.JPEG]
