# Supplementary material for: Generation of Realistic Gene Regulatory Networks by Enriching for Feed-Forward Loops
Source: Front Genet. 2022 Feb 10;13:815692. doi: 10.3389/fgene.2022.815692 (PMC8872634; doi:10.3389/fgene.2022.815692)
Supplement: Supplementary file 6 [file DataSheet1.docx]

***Supplementary Material***

# Supplementary Tables

**Table S1. Motif enrichment in Transcriptional interaction graphs**

|  | **Cascades** | | **Downlinks** | | **Uplinks** | | **Cycles** | | **FFLs** | |
| --- | --- | --- | --- | --- | --- | --- | --- | --- | --- | --- |
| **Organism** | Z-score | P-value | Z-score | P-value | Z-score | P-value | Z-score | P-value | Z-score | P-value |
| ***E.coli*** | -7.92 | <0.01 | -7.27 | <0.01 | -14.52 | <0.01 | -1.37 | 0.13 | **7.4** | 0 |
| ***S. cerevisiae*** | -1.88 | 0.04 | -1.94 | 0.03 | -13.49 | <0.01 | -0.68 | 0.32 | **9.11** | 0 |
| ***M. musculus*** | -5.66 | <0.01 | -5.20 | <0.01 | -9.42 | <0.01 | -1.83 | 0.03 | **4.92** | 0 |
| ***H. sapiens*** | -4.90 | <0.01 | -4.68 | <0.01 | -6.33 | <0.01 | -2.33 | <0.01 | **1.45** | 0.07 |

**Table S2. Motif enrichment generated with the FFLatt algorithm across parameter space (Z-score +-Se).** Motif enrichment analysis was calculated on networks of size of 500 and 750 nodes, ten of each size. The fraction of nodes that participate in FFL motifs in E.coli GRN was used to set *p1*, *p2* equal to (1-*p1*)**k*, and *p3* equal to 1-(*p1*+*p2*+*p4*).

| Motif depletion step | *k* | *p4* | **Cascades** | **Downlinks** | **Uplinks** | **Cycles** | **FFLs** |
| --- | --- | --- | --- | --- | --- | --- | --- |
| yes | 0.5 | 0.5 | -2.11±0.1 | -2.838±0.083 | -4.523±0.183 | -2.805±0.047 | **5.665±0.393** |
|  |  | 0.7 | -2.976±0.222 | -3.442±0.19 | -4.782±0.097 | -2.636±0.017 | **3.627±0.721** |
|  |  | 0.9 | -1.223±0.092 | -1.824±0.066 | -3.577±0.111 | -1.753±0.048 | **6.2±0.106** |
|  | 0.7 | 0.5 | -4.609±0.035 | -4.869±0.03 | -2.65±0.096 | -2.835±0.01 | **1.891±0.148** |
|  |  | 0.7 | -3.543±0.069 | -3.745±0.06 | -2.415±0.013 | -2.705±0.013 | **1.357±0.389** |
|  |  | 0.9 | -3.58±0.066 | -3.784±0.063 | -2.049±0.062 | -2.778±0.007 | **2.277±0.165** |
|  | 0.9 | 0.5 | -4.148±0.097 | -4.199±0.101 | -0.666±0.104 | -2.425±0.015 | **0.347±0.124** |
|  |  | 0.7 | -3.916±0.058 | -4.05±0.053 | -1.139±0.022 | -2.96±0.008 | **1.418±0.058** |
|  |  | 0.9 | -4.834±0.012 | -4.962±0.013 | -0.286±0.058 | -2.586±0.006 | **0.382±0.033** |
| no | 0.5 | 0.5 | 8.855±2.425 | 7.876±2.283 | -0.273±0.495 | 2.518±0.895 | **16.315±3.577** |
|  |  | 0.7 | 7.277±1.934 | 6.482±1.803 | -0.734±0.675 | -0.332±0.433 | **14.471±2.255** |
|  |  | 0.9 | 7.303±1.846 | 6.338±1.624 | -0.823±0.558 | -0.282±0.337 | **15.733±2.592** |
|  | 0.7 | 0.5 | 6.221±2.102 | 5.757±2.061 | 3.822±0.777 | 0.884±0.711 | **10.136±1.184** |
|  |  | 0.7 | 5.254±1.149 | 4.908±1.124 | 2.416±0.516 | -1.325±0.08 | **6.03±0.219** |
|  |  | 0.9 | 4.563±1.448 | 4.32±1.415 | 1.32±0.47 | -1.757±0.058 | **5.422±0.453** |
|  | 0.9 | 0.5 | 4.608±1.917 | 4.435±1.882 | 4.125±0.761 | -0.937±0.117 | **3.749±0.48** |
|  |  | 0.7 | 4.69±1.362 | 4.467±1.356 | 4.414±0.649 | -2.11±0.077 | **4.321±0.158** |
|  |  | 0.9 | 4.036±1.409 | 3.852±1.394 | 3.918±0.526 | -2.372±0.008 | **2.598±0.056** |

## Supplementary Figures

**Figure S1. Correlations between the degree of connected nodes, calculated as the weighted average nearest-neighbors degree, k_nn_(k)*,* in the original four real networks and after shuffling**. Mean k_nn_(k) values after 1000 shuffles are shown.

**Figure S2. Topological properties of simulated networks (*S. cerevisiae)*.** FFL-motif node participation, average sparsity, in- and out-degree distribution in simulated networks. For FLL-motif node participation counts, up to three participations for each node were allowed  (in different roles).

**Figure S3. Topological properties of simulated networks (*M. musculus)*.** FFL-motif node participation, average sparsity, in- and out-degree distribution in simulated networks. For FLL-motif node participation counts, up to three participations for each node were allowed  (in different roles).

**Figure S4. Topological properties of simulated networks (*H. sapiens)*.** FFL-motif node participation, average sparsity, in- and out-degree distribution in simulated networks. For FLL-motif node participation counts, up to three participations for each node were allowed  (in different roles).

**Figure S5. Mean runtime for FFLatt algorithm.** Ten datasets at each size were used for determining mean run time when generating networks with various numbers of FFL-motifs present.
